# Genetic and Phenotypic Characterization of Manufacturing Seeds for a Tetravalent Dengue Vaccine (DENVax)

**DOI:** 10.1371/journal.pntd.0002243

**Published:** 2013-05-30

**Authors:** Claire Y.-H. Huang, Richard M. Kinney, Jill A. Livengood, Bethany Bolling, John J. Arguello, Betty E. Luy, Shawn J. Silengo, Karen L. Boroughs, Janae L. Stovall, Akundi P. Kalanidhi, Aaron C. Brault, Jorge E. Osorio, Dan T. Stinchcomb

**Affiliations:** 1 Arboviral Diseases Branch, Division of Vector-Borne Diseases, Centers for Disease Control and Prevention, Fort Collins, Colorado, United States of America; 2 Inviragen Inc., Fort Collins, Colorado, and Madison, Wisconsin, United States of America; 3 Shantha Biotechnics Ltd., Hyderabad, India; Pediatric Dengue Vaccine Initiative, United States of America

## Abstract

**Background:**

We have developed a manufacturing strategy that can improve the safety and genetic stability of recombinant live-attenuated chimeric dengue vaccine (DENVax) viruses. These viruses, containing the pre-membrane (prM) and envelope (E) genes of dengue serotypes 1–4 in the replicative background of the attenuated dengue-2 PDK-53 vaccine virus candidate, were manufactured under cGMP.

**Methodology/Principal Findings:**

After deriving vaccine viruses from RNA-transfected Vero cells, six plaque-purified viruses for each serotype were produced. The plaque-purified strains were then analyzed to select one stock for generation of the master seed. Full genetic and phenotypic characterizations of the master virus seeds were conducted to ensure these viruses retained the previously identified attenuating determinants and phenotypes of the vaccine viruses. We also assessed vector competence of the vaccine viruses in sympatric (Thai) *Aedes aegypti* mosquito vectors.

**Conclusion/Significance:**

All four serotypes of master vaccine seeds retained the previously defined safety features, including all three major genetic loci of attenuation, small plaques, temperature sensitivity in mammalian cells, reduced replication in mosquito cell cultures, and reduced neurovirulence in new-born mice. In addition, the candidate vaccine viruses demonstrated greatly reduced infection and dissemination in *Aedes aegypti* mosquitoes, and are not likely to be transmissible by these mosquitoes. This manufacturing strategy has successfully been used to produce the candidate tetravalent vaccine, which is currently being tested in human clinical trials in the United States, Central and South America, and Asia.

## Author Summary

Transmitted by *Aedes* spp. mosquitoes found worldwide, dengue is the most important mosquito-borne viral disease in the world. The incidence of dengue has increased 30-fold over the past 50 years, and is now endemic in over 100 countries. Vaccination is believed to be one of the most effective strategies in dengue prevention. However, no vaccine is currently available, and prevention strategies to control mosquitoes in endemic areas have been insufficient in controlling dengue. We have developed a recombinant live-attenuated tetravalent vaccine against all four serotypes of dengue virus. This candidate vaccine is currently under human clinical evaluation. In this report, we provide information regarding our manufacturing strategy, and present details of the genetic and biological characterization of the master seed virus for each vaccine serotype. The study described here, our previously reported and ongoing pre-clinical studies, and current clinical trials will provide critical information to evaluate the safety and efficacy of the vaccine to protect humans against dengue.

## Introduction

The dengue virus (DENV) complex, genus *Flavivirus*, comprises four distinct serotypes of viruses (DENV-1 to -4). DENV causes the most rapidly spreading mosquito-borne human viral disease in the world. About 50–100 million new dengue infections occur each year in more than 100 endemic countries [1], with an estimated 500,000 cases exhibiting hemorrhagic manifestations [1], [2]. DENV infection can cause subclinical disease or overt illness ranging from mild dengue fever to life threatening dengue hemorrhagic fever (DHF) or dengue shock syndrome (DSS). Infection with one DENV serotype usually provides life-long immunity to the same serotype, but it does not elicit long-term cross-protective immunity against other serotypes. People with a previous DENV infection have a higher risk of developing more severe disease in a subsequent infection with a different serotype of DENV [3]. It has been shown that non-neutralizing cross-reactive antibodies bound to DENV enhances viral entry into Fcγ-receptor-bearing cells, resulting in increased virus load and/or production of certain cytokines that can increase viral infection and pathogenesis [4]. Therefore, the ideal DENV vaccine should provide simultaneous protection against all four serotypes of DENV.

We have developed a tetravalent recombinant live-attenuated dengue vaccine (DENVax) that is currently being tested in Phase 1 and Phase 2 human clinical trials. This vaccine consists of 4 serotypes of recombinant vaccine viruses, designated as DENVax-1, -2, -3, and -4. The preclinical research-grade viruses were developed using reverse genetic technology at the Division of Vector-Borne Diseases (DVBD), Centers for Disease Control and Prevention (CDC) [5]–[7], while the commercial-grade DENVax vaccine viruses were re-derived by Inviragen Inc. in collaboration with the CDC [7], in a cGMP facility at Shantha Biotechnics Ltd., Hyderabad. The infectious clone-derived DENVax-2 is based upon an attenuated DENV-2 strain, DEN-2 PDK-53 [8]–[11]. The DENVax-1, -3 and -4 are chimeric, recombinant viruses that were originally designated as D2/1-V, D2/3-V, and D2/4-V (research-grade) [6] and express DENV-1, -3, and -4 serotype-specific prM and E viral proteins, respectively, in the common genetic background of the attenuated DENVax-2. The major attenuation loci, nucleotide 5′NCR-57-T, NS1-53-Asp, and NS3-250-Val, of the DENV-2 PDK-53 vaccine have been previously determined, and all of them are shared by the common PDK-53 virus-specific genetic background of the four DENVax viruses [6], [12]. The genetic sequence of the three attenuation loci as well as the previously established *in vitro* and *in vivo* attenuation phenotypes of these vaccine candidates were carefully monitored for the cGMP-manufactured DENVax seeds. This report describes the strategies used to generate the master virus seeds (MVS) as well as their genetic and phenotypic characterization. These MVS can be used to manufacture clinical and ultimately commercial vaccine materials.

## Materials and Methods

### Ethics Statement

All animal experiments were conducted in accordance with the “Public Health Service Policy on Humane Care and Use of Laboratory Animals” by NIH, “Animal Welfare Act and Amendments” by USDA, “Guide for the Care and Use of Laboratory Animals” by National Research Council (NRC), “Occupational Health and Safety in Care and Use of Research Animals” by NRC, and “Biosafety in Microbiology and Biomedical Laboratories” by CDC. The animal experimental protocol was approved by the DVBD/CDC Institutional Animal Care and Use Committee.

### Viruses and Cells

DENV-1 16007, DENV-2 16681, DENV-3 16562, and DENV-4 1034 served as wild type (wt) DENV controls, and they were the parental genotype viruses for the DENVax viruses. DENVax progenitor research-grade viruses, designated as D2/1-V, D2 PDK-53-VV45R, D2/3-V, and D2/4-V, were prepared previously [6], [12]. Vero (African green monkey kidney) cells used for making the cell banks for vaccine production originated from the American Type Culture Collection CCL81 cell line that has been characterized by the World Health Organization (WHO) for manufacturing vaccines (WCB-Vero cells).

### Derivation of Live Recombinant DENVax Viruses from cDNA Clones

The engineered DENV infectious cDNA clones, pD2-PDK-53-VV45R, pD2/1-V, pD2/4-V, and *in vitro*-ligated pD2/3-V, containing the full genome-length viral cDNAs, were used to make fresh viral RNA transcripts by *in vitro* transcription as described previously [6], [12]. The RNA transcripts were treated with DNase I followed by low-pH phenol/chloroform extraction and ethanol precipitation to remove the template cDNA and proteins. Each sample was estimated to yield 2–4 ug of genome-length viral RNA that could produce over 5 log_10_ pfu/ml of the viruses after transfecting into 4×10^7^ Vero cells by electroporation using the Gene Pulser Xcell total system (BioRad Laboratories). Transfected cells were cultured in 30 ml of cell growth medium (MEM with 10% FBS), and incubated at 36°C±1°C, 5% CO_2_ for 6 to 11 days. These passage 1 (P1) virus seeds were harvested, clarified by centrifugation, stabilized, and stored in small aliquots below −60°C.

### Manufacture of DENVax Master Virus Seeds

The P1 virus seeds were used to propagate DENVax pre-master and master virus seed (MVS) lots through a strategy designed to ensure the optimal genetic stability and safety of the manufactured vaccines. This strategy included three serial plaque purifications, as well as genetic analyses of viruses to select the optimal clonal virus for continued seed production (Table 1). Briefly, the P1 seeds were amplified once in Vero cells at a MOI of 0.001 to generate the P2 seeds. The P2 seed stocks were evaluated by plaque morphology and complete viral genomic sequencing. The genetically confirmed P2 stocks were plated on Vero cells with overlay medium as described in the plaque assay below to generate well-isolated plaques, and six individual plaques from each serotype of DENVax were isolated (plaque clone A–F) and mixed into 0.5 ml of culture medium (P3). Each plaque suspension was subjected to two additional rounds of plaque purification, resulting in twice- and thrice-plaque purified virus seeds at passages P4 and P5, respectively. The P5 viruses were amplified through two sequential Vero passages to produce P7 seed stocks.

**Table 1:** cGMP Rederivation of DENVax MVS in WCB-Vero Cells.

Passage	Seed Production/Purification	Characterizations
P1	Transfect WCB-Vero with transcribed viral RNAs	Plaque titration
P2	Amplify P1 virus	Full genome sequence and plaque phenotype
P3	Pick 6 plaques (A–F)/serotype from P2 plaque assay	Plaque purification
P4	Pick plaques A–F from P3 plaque assay	Plaque purification
P5	Pick plaques A–F from P4 plaque assay	Plaque purification
P6	Amplify P5 A–F plaques	Plaque titration
P7	Pre-master seeds: Amplify P6 A–F	Full genome sequence, TaqMAMA, Plaque phenotypes
P8^a^	MVS: Amplify selected P7 virus seed	Full genetic and phenotypic characterization

a One optimal P7 seed (A, B, C, D, E, or F) was selected based on the genetic and plaque analysis to make the P8 MVS.

Genetic analysis of the three major DENVax attenuation loci [6], [12] and plaque phenotype analysis were conducted to screen all 24 P7 seeds. Seeds possessing appropriate initial characteristics were further characterized by full genomic sequencing. Based on the presence of the 3 major DENV-2 PDK-53 attenuating loci, minimal genomic sequence alterations, and expected plaque phenotype, one P7 clone of each serotype was selected to be the pre-master seed. The MVS (P8) was then generated by a one-passage amplification of the pre-master seed at MOI of 0.001 in multiple 175 cm^2^ flasks of Vero cells. The MVS stocks were harvested at 6–10 days post infection (pi), clarified by centrifugation, stabilized by the addition of serum or F127 (a pluronic block copolymer), trehalose and human albumin [13], and stored as 1-ml aliquots below −60°C.

### Virus Plaque Assay

Virus titers and plaque sizes were measured by plaque assay using Vero cells as previously described [5]. For accurate comparison, plaque sizes of all viruses were measured and compared in the same experiment. After visualization with neutral red on day 9 pi, up to 10 well isolated plaques for each virus were measured for mean plaque size calculation. Fewer plaques were measured for wt DENV-1, -3, and -4, whose larger plaque sizes often did not permit measurement of 10 well-separated plaques.

### Genetic Sequence and Taqman-based Mismatch Amplification Mutation Assay

Viral RNA was extracted from DENVax seeds and used for cDNA amplification as previously described [5], [6]. Automatic sequencing of the cDNA was conducted on the 3130xl Genetic analyzer (Applied Biosystems), and results were analyzed using Lasergene SeqMan software (DNAStar, Inc).

Taqman-based mismatch amplification mutation assay (TaqMAMA), a quantitative single nucleotide polymorphism assay, was previously developed to permit finer assessment of the level of reversion at the 5′NC-57 locus of attenuation [14], and was further optimized for this study. The specific forward primers used to detect DENV-2 wt and vaccine sequences were D2-41-GC and D2-40-TT, respectively. Triplicate reactions for each wt- and vaccine-specific assay were conducted for each sample, and specificity of the assay was confirmed by testing each RNA standard with the heterologous genotype primer/probe sets to ensure minimum cross-reactivity in every experiment. The real time RT-PCR was performed with the iQ5 or CFX-95 system (BioRad), using a BioRad iScript RT-PCR (for probes) kit. The results were reported as the percentage of viral genomes showing reversion. Previously, due to higher cross-reactive backgrounds that limited the input RNA levels for this assay, the detection sensitivity was at 0.1% reversion (discrimination power) [14]. In this study, the assay has been further optimized using improved equipment and reaction kits, and the cross-reactive background was decreased considerably at much higher levels (7–8 log_10_ copies) of RNA template input. This optimization resulted in significant improvement of the detection sensitivity, down to 0.01–0.07% reversion.

### Virus Replication in Mosquito C6/36 Cells and Temperature Sensitivity in Mammalian Vero Cells

The replication phenotypes of the DENVax MVS stocks were compared with their parental wt DENVs in C6/36 mosquito cells (*Aedes albopictus*) and Vero cells grown in 6-well plates. Cells were infected at a MOI of 0.001 and incubated with DMEM medium containing 2% FBS in a 5% CO_2_ incubator at 28°C (C6/36), or 37°C and 39°C (Vero). Aliquots of the culture supernatant were collected for each virus on day 5 (Vero) or 6 (C6/36) pi, mixed with an equal volume of medium containing 40% FBS, and stored at −80°C until processed by plaque titration. All samples were tested in duplicate for each experiment.

### Mosquito Infection, Dissemination, and Transmission

*Aedes aegypti* mosquitoes used for this study were from a colony established in 2002 from a village near Mae Sot (16′N, 33′E), Thailand. Five-to-seven day old female mosquitoes were used for infectious blood meal feeding or intrathoracic (IT) inoculations. Aliquots of freshly cultured DENVax and wt DENV were used immediately upon harvest (without any freeze-thaw cycle) to make virus blood meals, and the remaining virus suspensions were supplemented with 20% FBS and stored at −80°C for plaque titration and IT inoculation. The DENVax seeds for these experiments were prepared from one passage of the pre-master seeds in Vero cells, and were considered DENVax MVS equivalents.

Infectious blood meals were prepared by mixing fresh virus at a ratio of 1∶1 with defribrinated chicken blood (Colorado Serum Company). Mosquitoes were sugar-starved overnight and then offered the blood meal for 1 hour using a Hemotek membrane feeding system (Discovery Workshops). A 50-µl aliquot of the blood meal was retained at −80°C for back-titration of virus doses. Fully-engorged females were sorted under cold anesthesia and placed into cartons with 10% sucrose solution provided *ad libitum* at 28°C with a photoperiod of 16∶8 h (light∶dark). After 14 days, 25–30 mosquitoes from each group were anesthetized by triethylamine (Carolina Biological Supply Company) exposure, and one hind leg was removed and placed in 0.5 ml of DMEM with 10% FBS and penicillin/streptomycin (100 U/ml and 100 µg/ml respectively). Saliva was collected by inserting the proboscis of the mosquito into a capillary tube containing 2.5% FBS and 25% sucrose solution. Mosquitoes were allowed to salivate for at least 15 minutes and then capillary tubes and bodies were placed into separate tubes containing DMEM. For IT inoculation, mosquitoes were cold-anesthetized and inoculated with approximately 50 pfu of virus in 0.34 µl inoculum. Inoculated mosquitoes were kept for 7 days in the same conditions as described above. Mosquitoes were then anesthetized, and their saliva and bodies were collected as described above. All collected samples were stored at −80°C until they were further processed by plaque titration. Body and leg samples were homogenized with copper coated BBs (Crossman Corporation, NY) at 24 cycles/second for 4 min using a mixer mill, and then clarified by centrifugation. Saliva tubes were centrifuged at 3,000× g for 3 minutes to expel fluid from capillary tubes. Virus plaque titration results from bodies, legs, and saliva were used for determining the infection, dissemination, and transmission rates, respectively.

### Mouse Neurovirulence

Timed pregnant female ICR mice were obtained from Taconic Labs, and monitored for birth of pup litters. Approximately 12–24 hours after birth, two litters of eight pups per virus (n = 16) in each experiment, were challenged with 10^3^ to 10^4^ pfu of virus in 20 µl of diluent by intracranial (ic) inoculation using a 30-gauge needle. Animals were monitored at least 3 times daily for at least 32 days following challenge. At the first sign of illness (rough fur, hunched back, weight loss, abnormal movement, paralysis, or lethargy) animals were euthanized.

## Results

### Production and Analysis of Pre-master DENVax Viruses

DENVax viruses were re-derived under cGMP condition by transfection of viral RNA transcribed from the full-length recombinant cDNA into production-certified Vero cells, resulting in P1 virus seeds. The four P1 viruses were then amplified to obtain P2 seeds, which were subjected to full-length genome sequencing. Results showed that each of the four serotypes of P2 viruses was genetically identical to its homologous progenitor, research-grade candidate vaccine virus [6]. The original plaque phenotypes were also retained in the P2 viruses. Six plaque-purified viruses (A–F clones) were isolated for each serotype of DENVax from the P2 seeds, and each isolated plaque was directly plaque purified two more times. The third plaque purification (P5) of each virus was amplified twice in Vero cells to produce the potential pre-master P7 DENVax seeds (Table 1). Genome sequences and plaque phenotypes of the P7 seeds were analyzed and compared to the P2 seeds (Table 2). Plaque phenotypes of the P7 viruses were generally similar to those of the P2 seeds. One virus (DENVax-2 C) had somewhat larger than expected plaques, 2 viruses were smaller (DEVax-4 E and F), and 4 viruses (DENVax-1 C and D, DENVax-4 B and D) had slightly clearer plaques (Table 2). Virus titers reached over 6.0 log_10_ pfu/ml for most of the P7 seeds, except for 5 viruses.

**Table 2:** Characterizations of pre-master (P7) seeds.

Virus	Clone^a^	TaqMAMA^b^	Log_10_ pfu/ml	Plaque^c^	Mutations identified in genome^d^
DENVax-1	**A**	**	6.85	P2	NS2A-I116L, NS2B-E92D, one silent
	B	*	6.93	P2	nd^e^
	C	*	6.93	D	nd
	D	**	7.02	D	C-K67A; one silent
	E	0.57%	7.28	P2	nd
	F	**	7.18	P2	E-T473M; one silent
DENVax-2	A	0.03%	6.33	P2	NS1-K341N
	B	*	6.33	P2	E-K305T, two silent
	C	*	5.84	L	NS4A-T18A, four silent
	D	0.08%	6.20	P2	NS2B-I99L, one 3′NCR
	E	0.03%	6.31	P2	prM-K52E, NS5-I412V, two silent
	**F**	**	6.15	P2	prM-K52E, NS5-I412V
DENVax-3	A	*	6.00	P2	NS5-K200N, one silent, one 3′NCR
	B	0.05%	6.27	P2	NS2A-I33T, NS2A-M59T
	C	0.30%	6.25	P2	nd
	D	100.00%	6.27	P2	nd
	E	0.31%	6.00	P2	nd
	**F**	**	6.30	P2	E-T223S, one silent
DENVax-4	A	0.47%	5.60	P2	E-K323R/K, NS2B-L21F/L, NS2B-T39S, one silent
	B	*	5.65	D	NS2A-A126V; NS4A-N5D; NS5-K383R, one silent
	C	4.50%	5.90	P2	nd
	D	12.85%	5.97	D	nd
	E	0.52%	6.85	S	prM-E85D, NS2B-T45A, NS5-M320T, NS5-E551G, two silent
	**F**	0.02%	6.93	S	NS2A-D66G, NS4A-A21V, four silent

a Cloned viruses (by serial plaque purifications) selected for further development of MVS are designated in bold.

b *: Reversion rate <0.07% (detection limit).

** : Reversion rate <0.01% (detection limit).

c Plaque phenotypes: P2: similar to P2 virus; L = larger than P2 virus, D = similar size, but appear somewhat different in clearness of the plaques; S = smaller than P2.

d Substitutions differing from the engineered DENVax cDNA clones. Amino acid mutations are listed with residue position in the viral proteins. Total numbers of silent mutation in structural and non-structural genes, as well as substitution at non-coding regions (5′ or 3′NCR) are also noted.

e nd = Not done. These clones had higher 5′NCR-57 reversion rates (by TaqMAMA) than other clones, so were excluded from further sequence analysis.

We determined previously that two (NS1-53 and NS3-250) of the three major attenuation determinants of the DENV-2 PDK-53 genetic vector are extremely stable. Genome sequencing of more than 60 candidate vaccine virus seeds after 10 or more serial passages in Vero cells identified no reversion event at these 2 loci [[14] and unpublished data]. All sequence chromatograms generated from both forward and reverse sequencing for these two sites were very clean without any minor nucleotide populations evident at the NS1-53 and NS3-250 genetic loci. In contrast to the NS1 and NS3 sites, different levels of reversions at the 5′NCR-57 attenuation locus were previously identified from multiple serially passaged research-grade vaccine viruses [14], suggesting this locus might not be as stable as NS1 and NS3 after multiple passages in cell culture. Therefore, a sensitive TaqMAMA was developed to accurately measure the reversion rate at the 5′NCR-57 locus by real-time RT-PCR [14]. Depending on the concentration of the input viral RNA for each virus in the assay, the sensitivity of the assay ranged between 0.01% and 0.07% reversion, which is much more sensitive than the 10–30% reversion sensitivity limit detectable by consensus genome sequence analysis. We found 15 of the 24 viruses had minimal or undetectable reversion (<0.07%), 1 virus (DENVax-3 D) had almost 100% reversion, and 8 viruses (1 DENVax-1, 1 DENVax-2, 2 DENVax-3, and 4 DENVax-4) had partial reversion ranging from 0.08% to 12.85% (Table 2). Full-length genome sequencing was conducted for 16 of the 24 P7 viruses with low levels of 5′NCR57 reversion as measured by TaqMAMA. All of the sequenced viruses maintained the other 2 DENVax attenuation determinants at NS1-53 and NS3-250, and all had acquired additional mutations that were not present in the original, engineered recombinant cDNA clones (Table 2). DENVax-1 A, DENVax-2 F, DENVax-3 F, or DENVax-4 F was selected as the most optimal pre-master seed for each serotype because their genotypes and plaque phenotypes most closely resembled those of the originally designed vaccine viruses [6]. These pre-master seeds were further amplified to generate the MVS (Table 1).

### Genetic Analysis of DENVax MVS

Full-length genome sequencing revealed that the MVS for DENVax-1, DENVax-2, and DENVax-3 were identical to their respective pre-master seeds (Table 2 and 3). The DENVax-4 MVS acquired an additional amino acid mutation, at locus NS2A-K99K/R (from K to K/R mixed genotype) during production of the MVS. TaqMAMA results demonstrated that the 5′NCR-57 reversion rate was minimal or undetectable in all 4 MVS lots (Table 3).

**Table 3:** Nucleotide and amino acid substitutions in MVS.

DENVax (Clone)^a^	TaqMAMA^b^	Nucleotides^c^	Amino Acids^d^
DENVax-1 (A)	<0.07%	A3823C	NS2A-I116L
		A4407T	NS2B-E92D
		A7311G	silent
DENVax-2 (F)	<0.01%	A592G	prM-K52E
		A8803G	NS5-I412V
DENVax-3 (F)	<0.07%	A1603T	E-T223S
		A7620G	silent
DENVax-4 (F)	0.13%	A225T	silent
		A3674G	NS2A-D66G
		A3773A/G^e^	NS2A-K99K/R
		C5391T	silent
		C6437T	NS4A-A21V
		T7026C	silent
		A9750C	silent

a The DENVax was amplified from the indicated pre-master clone.

b Reversion rate at 5′NCR-57.

c Nucleotide position of the substitution and changes are indicated.

d Mutations resulting in amino acid changes are indicated.

e Mixed sequence signals were observed, indicating presence of both genotypes.

### Plaque Phenotype of DENVax MVS

**Figure 1:**
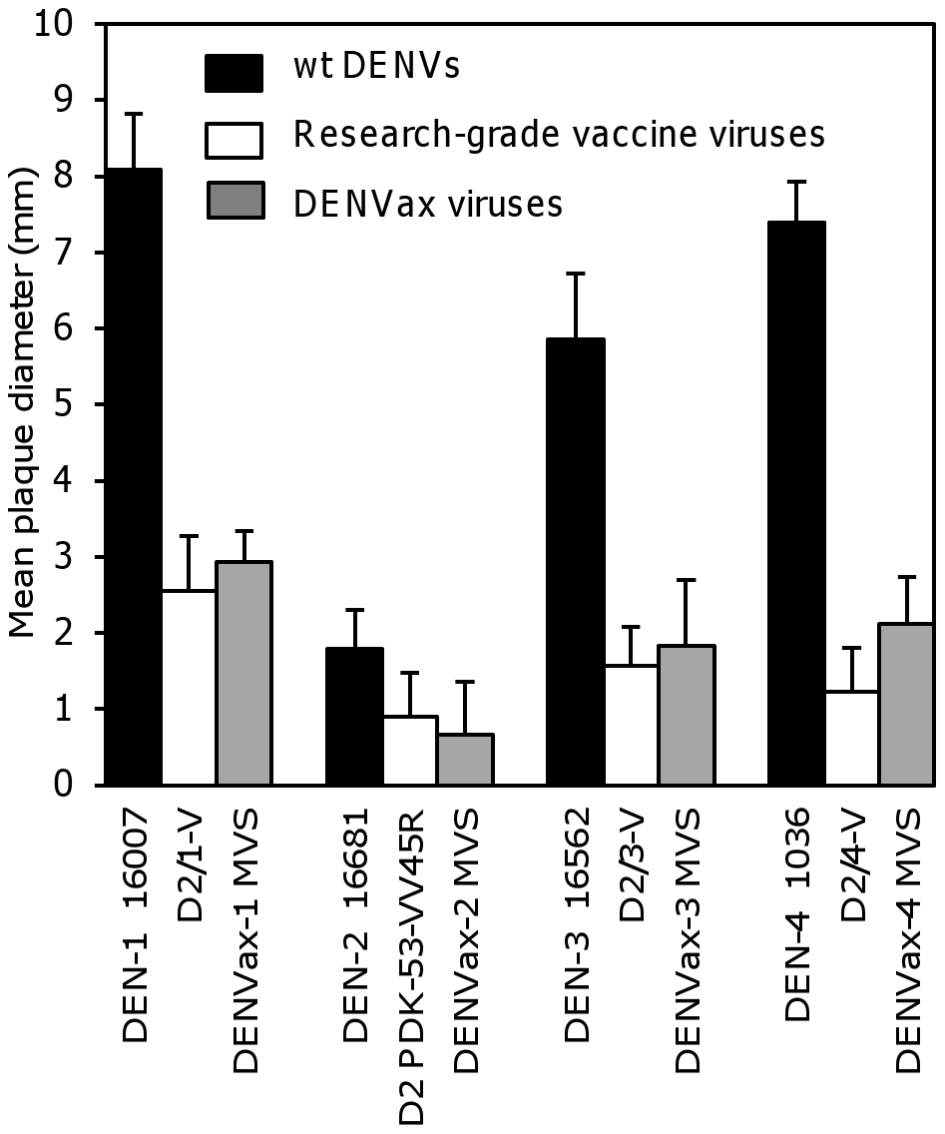
Plaque sizes of the DENVax MVS. Mean plaque diameters (mm) ± SD (error bars) of the virus plaques in Vero cells under agarose overlay measured on day 9 pi. The wt DENVs and previously published research-grade vaccine candidate viruses were included for control and comparison.

Plaque phenotypes of the DENVax MVS were compared with wt DENVs and their homologous research-grade chimeric viruses in Vero cells (Fig. 1). All of the MVS of DENVax-1, -2, and -3 produced plaques that were significantly smaller than their wt homologs and very similar (within 0.4-mm differences) to their homologous research-grade viruses in Vero cells. DENVax-4 MVS was also significantly smaller than the wt DENV-4, but was slightly larger (0.9 mm difference) than the original research-grade D2/4-V chimera.

### Temperature Sensitivity of DENVax MVS

**Figure 2:**
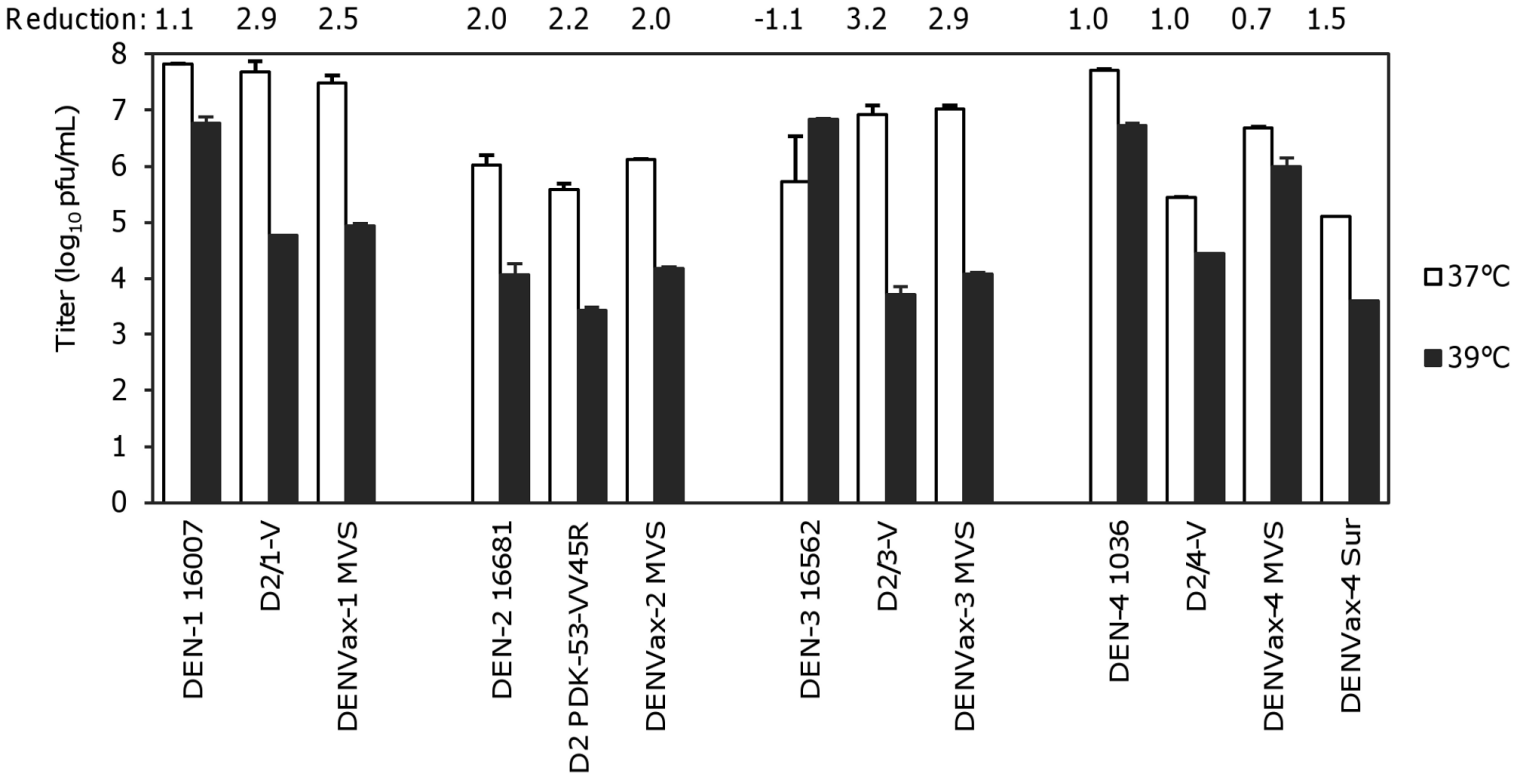
Temperature sensitivities of DENVax MVS. Mean titers ± SD (error bars) of the viruses replicated in Vero cells at 37°C or 39°C. The wt DENVs and previously published research-grade vaccine candidate viruses were included for comparison. The DENVax-4 MVS contains additional F-127 that can mask the temperature sensitivity results of the virus in this assay. A separate experiment analyzing a surrogate DENVax-4 in the absence of F127 was also included.

Temperature sensitivity was tested in Vero cells for the DENVax MVS and compared with their homologous wt and the original research-grade chimeric vaccine virus. The wt DENV-3 16562 was not temperature sensitive. The wt DENV-1 and DENV-4 were moderately temperature sensitive at 39°C (titers were approximately 1.0 log_10_ pfu/ml lower at 39°C than at 37°C, Fig. 2). Wt DENV-2 16681 was the most temperature sensitive of the wt DENVs tested, and resulted in a 100-fold titer drop at 39°C. DENVax-1, -2, and -3 were as temperature sensitive as their original homologous research-grade chimeric vaccine viruses (Fig. 2). Titers at 39°C dropped between 2.0 and 3.0 log_10_ pfu/ml for these DENVax strains. DENVax-4 was also temperature sensitive, demonstrating a 5-fold reduction in titer. However, the original research-grade D2/4-V showed about 10-fold reduction in titer. The final stabilized DENVax-4 MVS contained pluronic block copolymer F127, and we have previously shown that this copolymer can enhance thermal stability of the DENVs [13]. The presence of the copolymer F127 in DENVax-4 MVS likely contributed to the less pronounced temperature sensitivity of the virus in the Vero culture assay. In a separate experiment, we further evaluated the temperature sensitivity of an MVS-derived DENVax-4 strain in the absence of F127. To remove the F127 from the strain, viral RNA was isolated from a DENVax-4 bulk virus preparation and transfected into Vero cells. This DENVax-4 virus appeared to be as temperature sensitive as the D2/4 V (titer reduced 1.5 log_10_ pfu/ml) on day 3 pi in the absence of F127 (Fig. 2).

### DENVax MVS Replication in Mosquito C6/36 Cells

**Figure 3:**
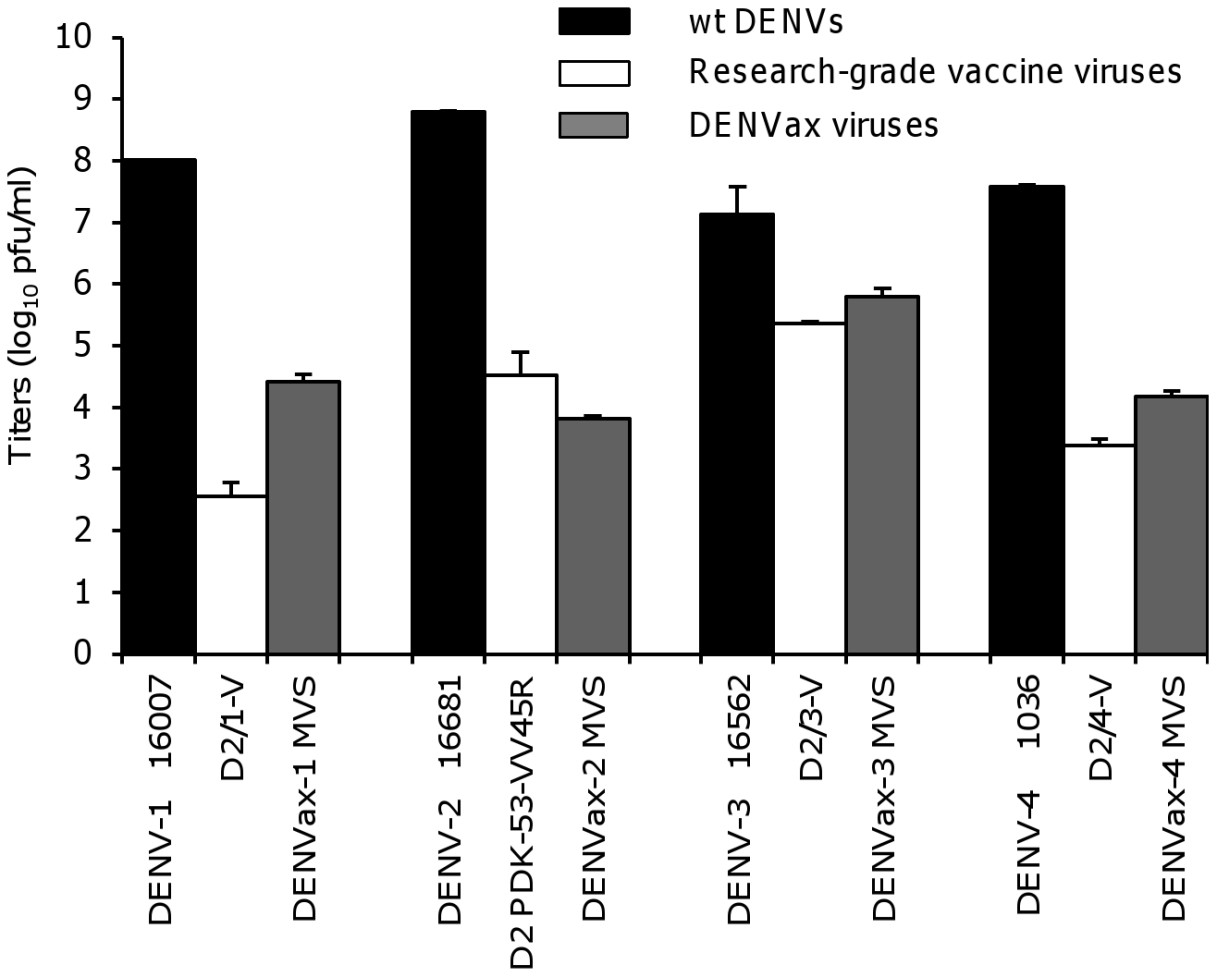
Restricted growth of DENVax MVS in C6/36 cells. Mean titers ± SD (error bars) of the viruses replicated in C6/36 cells 6 days pi. The wt DENVs and previously published research-grade vaccine candidate viruses were included for comparison.

Previous studies showed that the research-grade chimeric vaccine viruses retained the attenuation phenotype of the backbone DENV-2 PDK53 virus in C6/36 cells [6], [15]. The DENVax MVS were grown in C6/36 cells to verify their retention of this *in vitro* attenuation phenotype. Compared to the wt DENVs, DENVax-1, DENVax-2, and DENVax-4 MVS showed marked growth reduction (at least 3 log_10_ pfu/ml reduction) in C6/36 cells on day 6 pi (Fig. 3). The DENVax-3 MVS also exhibited reduced growth compared to the wt DENV-3 16562, but the reduction was not as marked (1–2 log_10_ pfu/ml reduction). However, the titer of the DENVax-3 was similar (within 1 log_10_ pfu/ml difference) to the titer of the research-grade chimeric D2/3-V vaccine virus.

### Virus Infection, Dissemination, and Transmission Rates in Whole Mosquitoes

Oral infection experiments were conducted in *Ae. aegypti* mosquitoes to evaluate the ability of DENVax to infect midgut (overcome midgut infection barrier; MIB), disseminate to secondary tissues (overcome midgut escape barrier; MEB), and be transmittable (release virus to saliva). Infectious blood meals were back-titrated to measure the virus titers and only the experiments with similar virus titers in the blood meal (less than 1 log_10_ pfu/ml differences) between parental DENV and DENVax for each serotype were included for comparisons in Table 4. DENVax-1, DENVax-2, and research-grade D2 PDK-53-VV45R did not infect mosquitoes through oral feeding, which was significantly different (p<0.0001) from their parental viruses, DENV-1 16007 (44% infection) and DENV-2 16681 (43.3% infection). Since no mosquito was infected by DENVax-1 and -2, there was no dissemination concern for these two vaccine viruses. While DENVax-4 did infect 2 of the 55 mosquitoes through oral feeding, the infection rate was significantly lower (p<0.05) than the wt DENV-4 1036. DENVax-3 did not infect any mosquitoes in two experiments with blood meal viral titers of 5.2±0.02 log_10_ pfu/ml (Table 4), and in a separate experiment with blood meal viral titer of 6.0 log_10_ pfu/ml, only 1 out of 30 mosquitoes became infected (data not shown in Table 4). However, wt DENV-3 16562 also had a very low infection rate (8%) at 5.2 log_10_ pfu/ml, and the rate did not increase in a separate experiment with a higher blood meal viral titer at 6.2 log_10_ pfu/ml (3%, 1 positive out of 30 mosquitoes). Although the wt DENV-3 and DENV-4 had significantly lower infection rates than the wt DENV-1 and DENV-2, the mean virus titers in the infected mosquitoes were similar (3.1 to 3.9 log_10_ pfu/mosquito). In contrast, the DENVax-4 titers from the two infected mosquitoes were both minimal (0.7 log_10_ pfu/mosquito), which was 1,000-fold lower than the titer from the mosquitoes infected by wt DENV-4 1036 (3.9±1.5 log_10_ pfu/mosquito). For those mosquitoes that were infected, dissemination out of the midgut could be assessed by determining whether virus was present in the legs. The four wt DENVs resulted in dissemination rates ranging between 36.3% and 62.5%, and their mean virus titers (in log_10_ pfu) from the legs were between 0.9±0.3 and 2.2±0.7 (excluding negative samples). Neither of the two DENVax-4 infected mosquitoes resulted in virus dissemination to the legs (Table 4).

**Table 4:** Virus infection, dissemination, and transmission rates in whole mosquitoes.

	Oral Feed					IT inoculation				
	Blood Meal^a^	Infection^b^	Body Titer^c^		Dissemination^e^	Inoculum	Infection^b^	Body Titer^c^	Saliva^f^	
Virus	Mean±SD	% (P/N)	Mean±SD	*p* d	% (P/N)^f^	pfu/dose	% (P/N)	Mean±SD	% (P/N)	*p* d
DENV-1 16007	6.6	44.0% (11/25)	3.6±1.5		36.3% (4/11)	53.9	100% (30/30)	4.7±0.48	43% (13/30)	
DENVax-1	6.9	0% (0/30)	NA	<0.0001	NA	67.8	100% (30/30)	3.4±0.39	10% (3/30)	<0.005
DENV-2 16681	6.6	43.3% (13/30)	3.1±1.5		38.5% (5/13)	67.8	100% (30/30)	5.2±0.34	87% (26/30)	
D2 PDK53-VV45R	6.4	0% (0/30)	NA	<0.0001	NA	56.4	100% (30/30)	4.0±0.20	0% (0/30)	<0.0001
DENVax-2	6.4	0% (0/30)	NA	<0.0001	NA	52.7	100% (30/30)	3.5±0.27	7% (2/30)	<0.0001
DENV-3 16562	5.2	8% (2/25)	3.8±0.2		50% (1/2)	34.0	100% (30/30)	4.2±0.50	67% (20/30)	
DENVax-3	5.2±0.02	0% (0/50)	NA	0.108	NA	37.3	100% (30/30)	3.3±0.36	3% (1/30)	<0.0001
DENV-4 1036	5.8±0.5	16% (8/50)	3.9±1.5		62.5% (5/8)	69.4	100% (30/30)	5.2±0.45	70% (21/30)	
DENVax-4	5.4±0.4	3.6% (2/55)	0.7±0.0	0.033	0% (0/2)	11.8	70% (21/30)	1.1±0.46	0% (0/21)	<0.0001

a Virus titers or Mean±standard deviation if from more than 1 experiment in blood meal (log_10_ pfu/ml) by back titration.

b Rate of virus detected in mosquito bodies. P/N = positive/total mosquitoes.

c Mean virus titers ± standard deviation (log_10_ pfu/mosquito) in mosquito body. Only positive samples are included for calculation.

d Statistical analysis of the differences between wt DENV and DENVax by Fisher Exact probability.

e Rate of virus detected in legs of the positively infected mosquitoes.

f Rate of virus detected in saliva of the positively infected mosquitoes. Used to measure transmission efficiency.

Since the oral feeding results showed minimal or no detectable midgut infection and body dissemination by DENVax, we could not evaluate their transmission potentials from this experiment. In addition, while disseminated virus was detectable in the legs, none of the four wt DENVs was detectable in saliva of orally infected mosquitoes (not shown in Table 4) which suggested that the oral feeding experiment may not be sufficiently sensitive to measure the transmission rate of these DENVs. Therefore, highly stringent artificial infections by IT inoculation were performed to directly infect the mosquito body bypassing both MIB and MEB. Except for DENVax-4, all viruses (wt and DENVax) achieved 100% infection of the IT inoculated *Ae. aegypti*. The DENVax-4 inoculum had a slightly lower viral titer than the other three viral inocula, but it still successfully infected 70% of the inoculated mosquitoes (Table 4).

Despite the high body infection rates achieved by IT inoculation, all four DENVax viruses exhibited significantly lower (p<0.005) or non-detectable transmission rates (0–10%) compared to the wt DENVs (43–87%, Table 4). The mean expectorate virus titers calculated from saliva (positive samples only) were slightly higher from wt DENV infected mosquitoes (0.92±0.75 log_10_ pfu, 0.88±0.31, 0.96±0.60, and 0.86±0.63, for wt DENV-1 to -4, respectively) than those infected with DENVax (0.40±0.00, 0.55±0.21, 0.88 from only 1 mosquito, and none detectable, for DENVax-1 to -4, respectively). Overall, the DENVax viruses showed no or limited infection and dissemination after oral feeding, and the highly stringent IT results affirmed the minimal transmission capacity of these DENVax viruses in *Ae. aegypti*.

### Neurovirulence in Newborn Mice

**Figure 4:**
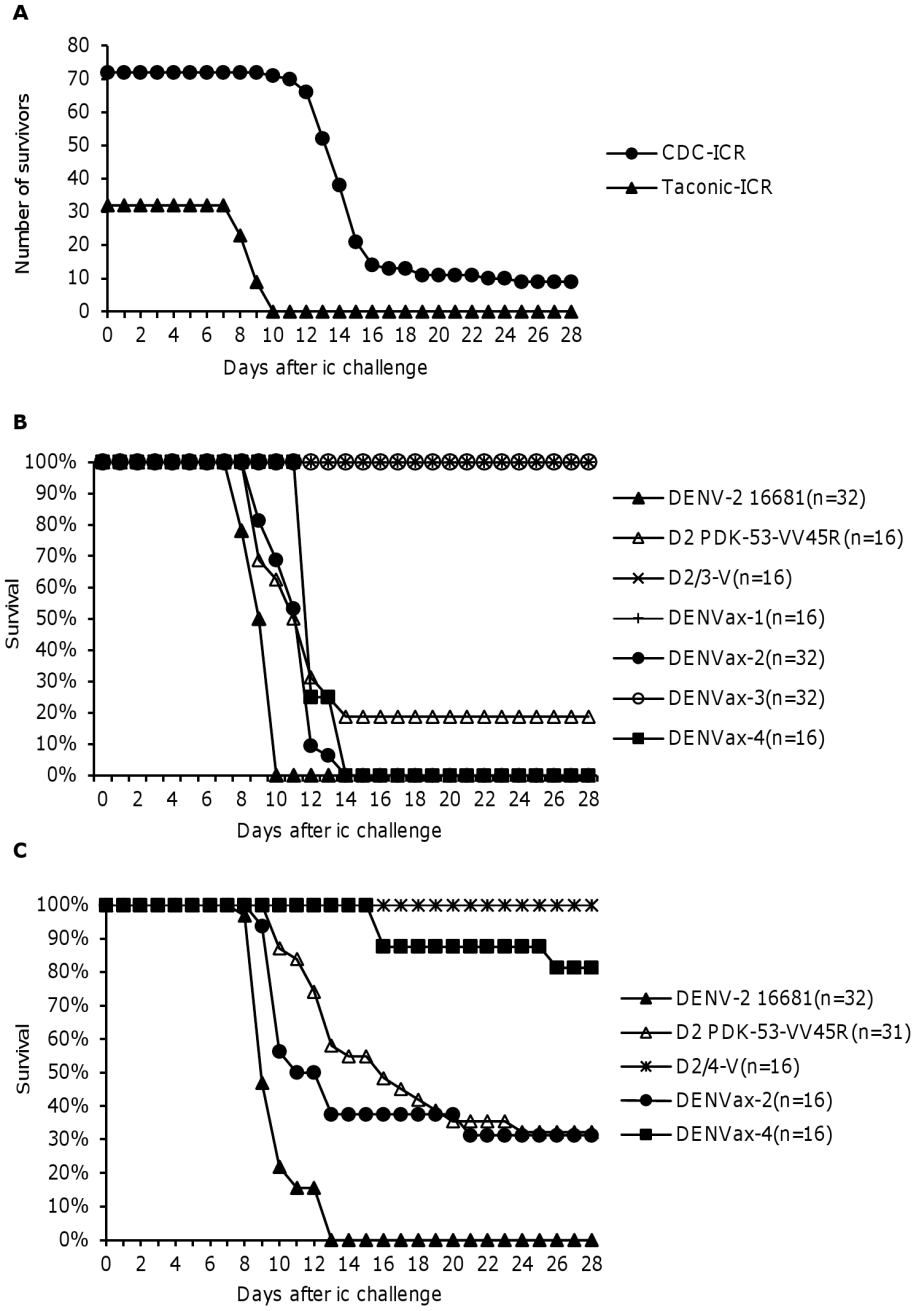
Neurovirulence in newborn mice. Pooled results of numerous experiments summarizing the neurovirulence of wt DENV-2 16681 virus in CDC-ICR (*n* = 72) and Taconic-ICR (*n* = 32) newborn mice challenged ic with 10^4^ pfu of the virus (A). Neurovirulence of DENVax MVS tested in Taconic-ICR mice with a dose of 10^4^ pfu (B) or 10^3^ pfu (C). The numbers of animals tested per group in one experiment (n = 16) or two pooled experiments (n = 31 or 32) are indicated.

The research-grade vaccine viruses were previously shown to be highly attenuated for neurovirulence in newborn ICR mice maintained in-house at DVBD/CDC [5], [6]. All of these mice survived ic challenge with 10^4^ pfu of each vaccine virus. The wt DENV-2 16681 virus, on the other hand, resulted in 62.5%–100% mortality in these CDC-ICR mice in various experiments. Prior to the current study the in-house ICR mouse colony was eliminated at DVBD/CDC. Therefore, commercial ICR mice obtained from Taconic Labs (Taconic-ICR) were used for this study. We observed that newborn Taconic-ICR mice were significantly more susceptible to DENV-2 infection than the previous CDC-ICR mice. Figure 4A summarizes the neurovirulence of wt DENV-2 16681 virus in CDC-ICR colony (previous published and unpublished experiments) and Taconic-ICR newborn mice challenged ic with 10^4^ pfu of virus. Clearly, the Taconic-ICR mice (100% mortality in 32 mice, average survival time of 8.3±0.5 days) were much more susceptible to ic DENV-2 16681 challenge than the previous CDC-ICR mice (91% mortality in 72 mice, average survival time of 14.6±2.3 days).

To evaluate neurovirulence of the DENVax MVS, the Taconic-ICR mice initially were challenged ic with a dose of approximately 10^4^ pfu of wt DENV-2 16681, D2 PDK-53 VV45R, D2/3-V, or DENVax 1–4 virus in one (n = 16) or two (n = 31–32) experiments (Fig. 4B). At this dose, D2/3-V research-grade virus, as well as DENVax-1, and DENVax-3 MVS exhibited fully attenuated neurovirulence phenotypes (no illness or mortality). As expected, wt DENV-2 was uniformly fatal, with average mouse survival time (AST) of 8.8±0.4 days. In these DENV-2-sensitive Taconic-ICR mice, the D2 PDK-53-VV45R research-grade virus resulted in 81.3% mortality with AST of 11.1±1.1 days. The DENVax-2 MVS and DENVax-4 MVS were uniformly fatal in the Taconic-ICR, showing AST values of 11.7±1.1 and 11.5±0.9 days, respectively. The mortality and AST between the D2 PDK-53-VV45R and DENVax-2 at this dose were not significantly different (p>0.05, student t-test). The research-grade D2/4-V was not tested at this dose for comparison with DENVax-4.

In an attempt to identify a more discriminating ic challenge dose for those viruses that exhibited neurovirulence in the Taconic-ICR mice, we compared the neurovirulence of wt DENV-2 16681 virus with that of D2 PDK-53-VV45R, DENVax-2 MVS and DENVax-4 MVS, as well as D2/4-V research-grade virus, at a 10-fold lower dose (10^3^ pfu, Fig. 4C). The wt DENV-2 retained a uniformly fatal neurovirulent phenotype, with AST of 9.0±1.4 days, at this lower challenge dose. The other 4 viruses exhibited intermediate neurovirulence phenotypes, and the degree of neurovirulence was serotype-specific. The D2 PDK-53-VV45R virus and its DENVax-2 MVS cognate showed significant attenuation (32.3% survival with AST of 13.8±3.6 days and 31.2% survival with AST of 11.5±3.4 days, respectively) and the differences were not significant (p = 0.4). Both the DENVax-4 MVS and the research-grade D2/4-V virus were highly attenuated for neurovirulence (81.3% survival with AST of 19.4±5.8 days and 100% survival, respectively). The slightly higher virulence of the DENVax-4 relative to D2/4-V was likely due to the higher virus dose that was inoculated into the mice receiving DENVax-4 (3.4 log_10_ pfu) versus the dose in the D2/4-V group (2.7 log_10_ pfu), based on the back titration results of the inocula. Overall, our results indicated that MVS of DENVax-1 and -3 exhibited complete attenuation of neurovirulence, while DENVax-2 and -4 MVS lots retained attenuation phenotypes that closely resembled their homologous research-grade vaccine candidates.

## Discussion

This report describes the process of re-deriving and preparing the DENVax manufacturing seed strains under cGMP conditions, and the genetic and phenotypic characterizations of the MVS. Serial plaque purifications and full-genome sequence analyses were incorporated into the manufacturing process to ensure production of vaccine seeds with optimal safety and genetic stability. All four of the chosen pre-master seeds had undetectable reversion at any of the 3 attenuation loci, contained one or two new amino acid substitutions in the viral translated proteins, and retained the small plaque phenotypes of the previous research-grade vaccine viruses. All of the DENVax viruses also were tested for identity, sterility, and lack of detectable adventitious agents as part of the manufacturing product release (data not shown). Full-genome sequence analysis revealed that an additional amino acid mutation evolved in the DENVax-4 MVS versus its pre-master seed, while the other three DENVax MVS lots retained the consensus genome sequence of their pre-master seeds. Overall, from derivation of the P1 seeds to the pre-master (P7) seeds, only 1 or 2 non-synonymous mutations occurred in a given seed. From P1 to MVS (P8) seeds, 2 to7 nucleotide substitutions were identified in any given DENVax seed and only 2 to 3 of these substitutions resulted in amino acid changes. RNA viruses are error-prone in their genome replication, so genetic substitutions in the flavivirus genome during cell passages are not unexpected. None of the silent mutations in the MVS were within the 5′ or 3′NCR that may affect virus replication. Most of the amino acid substitutions were very conservative (similar in residue size, pKa, and chemistry structure). Only the change in prM-K52E of the DENVax-2, and the substitution in NS2A-D66G of DENVax-4 were non-conservative changes. The NS2A-D66G mutation of the DENVax-4 resides in the nonstructural gene region of the shared DENV-2 PDK-53 genetic background. Although the NS2A-66 locus is usually D among various strains of DENV-2, interestingly it is usually G for DENV-4. It is possible that the D to G change in the DENVax-4 is important for fitness of the DENVax-4 in Vero cells. The DENVax-2 prM-K52E mutation resides in the C-terminal portion of the prM that is cleaved out from the mature virus particles. Overall, our phenotypic characterization results confirmed that none of the mutations in the MVS seeds significantly altered the attenuation phenotypes of the candidate vaccine viruses.

Our results suggested that the DENVax viruses are genetically stable during the manufacturing process. The highly sensitive TaqMAMA of the 5′NCR-57 locus demonstrated minimal or undetectable reversion in the MVS of each DENVax serotype. The 5′NCR-57 reversion rates of the DENVax MVS (P8) were significantly lower than the 5′NCR-57 reversion rates that evolved in research-grade vaccine candidates after 8-serial passages in Vero cells (6–45% reversion) [14]. The retention of the 3 attenuation loci were also confirmed in the P10 seeds that were passaged twice from the MVS (data not shown). Thus, the strategy for large-scale manufacturing of the DENVax seeds presented in this report resulted in genetically stable vaccine seeds which retained the expected markers of attenuation.

Complete evaluation of the phenotypic markers of viral attenuation, including small plaque phenotype, temperature sensitivity, reduced replication in mosquito cells, reduced infection/dissemination/transmission by mosquitoes, and attenuation in newborn ICR mice, were assessed for the MVS stocks. Although we cannot totally rule out that the slight phenotype variations between the DENVax and original research vaccine viruses were caused by the additional mutations that evolved in DENVax MVS stocks, none of the variations was very significant and all of the DENVax were still considerably attenuated relative to their wt counterparts. It will be important to analyze the clinical study outcomes in the context of genome sequences of clinical vaccine lots, and perhaps, viruses isolated from vaccinees to fully evaluate the safety and genetic stability of DENVax in humans.

Vector competence is an important safety component for live-attenuated flavivirus vaccine viruses [16]–[21]. We have previously tested the research-grade DENV-2 PDK-53-VV45R virus and wt reversion mutant derivatives in *Ae. aegypti*, and found that the NS1-53-Asp attenuating mutation was the dominant determinant for impaired replication in the mosquito [22]. The other two major attenuation loci of the DENV-2 PDK-53 vaccine, nucleotide 5′NCR-57-T and NS3-250-Val, also exhibited some inhibiting effect on replication in mosquitoes, thus providing additional, redundant restrictions for mosquito vector competence. This study is the first to report the mosquito oral and IT infection and replication for all four DENVax strains. DENVax-1, -2, and -3 did not infect any *Ae. aegypti* mosquitoes through oral infection (Table 4). The DENVax-4 infected only 3.6% of orally exposed mosquitoes, a level significantly lower than that of the wt DENV-4 with a replicative mean titer in the mosquito bodies lower than that of wt DENV-4 infected mosquitoes. Most importantly, no DENVax-4 was detected in the legs of the infected mosquitoes, suggesting that DENVax-4 was not able to escape from the midgut barriers following oral infection. The infection rates for the DENVax-1, -2, and -4 were all significantly less than their wt counterparts, but the difference was not significant between DENVax-3 and wt DENV-3 16562 due to the very low infection rates for both viruses. Compared to other wt strains of DENV assessed in *Ae. aegypti* collected from the same Mae Sot Province, Thailand [21], the parental wt DENV strains used for engineering our DENVax appeared to have lower infection and dissemination rates following oral exposure. The wt DENV-1 PUO359, DENV-2 PUO218, DENV-3 PaH881/88, and DENV-4 1288 used for engineering the yellow fever virus (YFV) 17D vaccine-based ChimeriVax-DEN vaccines had infection rates ranging 47–77%. In contrast, the YFV 17D vaccine cannot infect *Ae. aegypti* [21]. Although the ChimeriVax strains contained the prM-E from these highly infectious wt DENV, the ChimeriVax retain the mosquito attenuation phenotype of their YFV 17D replicative backbone [21]. Our results also indicated that the mosquito attenuation phenotype of the DENV-2 PDK-53 backbone was maintained in the DENVax strains. In addition, using the wt DENV strains with lower mosquito-infectivity in our constructs provides an additional safety feature for DENVax.

The oral infection results showed clearly that the DENVax had minimum mosquito infectivity and dissemination capacity. In addition, we performed the more sensitive IT inoculation to infect the mosquito body directly. This allowed us to establish a model to evaluate the potential of transmission if, by rare chance, any of the DENVax were able to overcome both MIB and MEB. Our results demonstrated that all four DENVax viruses had non-detectable or minimal mosquito transmission potential compared to their wt counterparts even with such an artificially high infection rate. DENVax transmission could only theoretically occur if (1) a vector feeds on a vaccinee having a sufficient viremic titer to infect mosquito midgut epithelium; (2) the virus is capable of replicating in these cells and subsequent dissemination out of the midgut; and (3) the disseminated virus can replicate in salivary gland and expectorate sufficient virus in saliva for transmission. The threshold of human viremia required to infect mosquitoes has not been established adequately, but human viremia can be 10^6^–10^8^ mosquito infectious dose_50_ (MID_50_)/ml after natural wt DENV infection [23], [24]. This MID_50_ was based on direct IT inoculation of mosquitoes with diluted human plasma. A previous study analyzing DENVax in nonhuman primates (NHP) indicated that viremic titers following DENVax immunization were very low (less than 2.4 log_10_ pfu/ml) and lasted for 2–7 days [25]. Given the low viremia levels and the low mosquito infection, dissemination, and transmission capacity of DENVax, it is unlikely that these vaccine viruses could be transmitted by mosquitoes in nature.

Unlike some other flaviviruses, such as Japanese encephalitis virus and YFV that can cause encephalitis in humans and show neuropathic effects in the NHP neurovirulent test, wt DENVs are generally not neurotropic in humans and the NHP [26], [27]. Previous neurovirulence studies of DENVs, including the wt DENV-1 16007, DENV-2 16681, DENV-3 16562, and DENV-4 1036 that were the parental viruses for engineering the 4 DENVax strains, showed that they caused only minimal neuropathophysiology after intracerebral injection into NHP [26], [28]–[31]. In addition, the DENVax seeds were not generated in a manner that could enhance neurovirulence; thereby they would not be expected to be neuropathologic in a NHP test. Infant mice have been demonstrated to be an accurate surrogate for the neurovirulence test of DENV vaccines in NHP previously [32], and the newborn mouse neurovirulence model reported in this study provided further support that certain young suckling mice can be a sensitive model for DENV vaccine evaluation.

In summary, we have successfully developed a unique manufacturing strategy to optimize the genetic stability and safety of the manufactured DENVax MVS. Since the main attenuation loci of the DEN-2 PDK-53-based DENVax have been well characterized previously [6], [12], we were able to integrate genome sequence and the TaqMAMA to identify optimal pre-master seeds for preparation of the MVS. Our results highlight the advantage of employing strategically designed live-attenuated vaccines with well-defined molecular attenuation determinants in predicting vaccine safety. In addition to the attenuation characteristics analyzed and described in this report, we have tested and confirmed the safety and immunogenicity of the DENVax MVS in AG129 mice [33] and NHP [25]. The present study described here and the ongoing clinical trial studies of DENVax will provide critical information to further evaluate the safety and efficacy of tetravalent DENVax as a vaccine to protect humans against dengue.
